# The association of estimated cardiorespiratory fitness with COVID-19 incidence and mortality: A cohort study

**DOI:** 10.1371/journal.pone.0250508

**Published:** 2021-05-05

**Authors:** Rebecca A. G. Christensen, Jasleen Arneja, Kate St. Cyr, Shelby L. Sturrock, Jennifer D. Brooks

**Affiliations:** Division of Epidemiology, Dalla Lana School of Public Health, University of Toronto, Toronto, Canada; Cincinnati Children’s, UNITED STATES

## Abstract

**Background:**

It has been suggested that cardiorespiratory fitness (CRF) may be used to identify those at greatest risk for severe COVID-19 illness. However, no study to date has examined the association between CRF and COVID-19. The objectives of this study were to determine whether CRF is independently associated with testing positive with or dying from COVID-19.

**Methods:**

This is a prospective cohort study of 2,690 adults from the UK Biobank Study that were followed from March 16^th^, 2020 to July 26^th^, 2020. Participants who were tested for COVID-19 and had undergone CRF assessment were examined. CRF was estimated (eCRF) and categorized as low (<20^th^ percentile), moderate (20^th^ to 80^th^ percentile) and high (≥80^th^ percentile) within sex and ten-year age groups (e.g. 50–60 years). Participants were classified as having COVID-19 if they tested positive (primarily PCR tests) at an in-patient or out-patient setting as of July 26, 2020. Participants were classified as having died from COVID-19 if the primary or underlying cause of death was listed ICD-10 codes U071 or U072 by June 30^th^, 2020. Adjusted risk ratios (aRR) and 95% confidence intervals (CI) were estimated and a forward model building approach used to identify covariates.

**Findings:**

There was no significant association between eCRF and testing positive for COVID-19. Conversely, individuals with moderate (aRR = 0.43, 95% CI: 0.25, 0.75) and high fitness (aRR = 0.37, 95% CI: 0.16, 0.85) had a significantly lower risk of dying from COVID-19 than those with low fitness.

**Conclusions:**

While eCRF was not significantly associated with testing positive for COVID-19, we observed a significant dose-response between having higher eCRF and a decreased risk of dying from COVID-19. This suggests that prior gains in CRF could be protective against dying from COVID-19 should someone develop the virus.

## Introduction

Severe acute respiratory syndrome coronavirus 2 (SARS-CoV-2) is a novel virus that was first detected in December 2019 [1] and has rapidly evolved into a global pandemic. As of September 9, 2020, there were 27,628,190 reported cases of coronavirus-disease 19 (COVID-19) worldwide, with 898,757 reported deaths [2].

Physical inactivity is an area of public health concern. Research suggests that physical inactivity is associated with increased COVID-19 severity [3]. Regular physical activity can improve overall health by decreasing the incidence [4–9] and morbidity [3, 5, 10] of chronic and communicable diseases. However, because the greatest gains are from physical activity that was sustained over a period of time [6, 10], the assessment of episodic physical activity may not be the best indicator of heath outcomes.

Cardiorespiratory fitness (CRF) is the ability of the body to supply oxygen to skeletal muscles during sustained activity and increases with regular physical activity [11]. CRF is an objective, reproducible measure that captures the health benefits of sustained physical activity [12]. CRF is also an established predictor of cardiovascular disease [13–15] and all-cause mortality [13–15], independent of age and body mass index—risk factors associated with COVID-19 severity and mortality. In fact, CRF has been found to better predict heart disease than assessments of physical activity, suggesting that it may be the better assessment of being active [16]. As such, several groups have hypothesized that high CRF may reduce the risk, severity, and duration of viral infections, including COVID-19 [17, 18]. However, to date, no study has examined the association between CRF and COVID-19 infection or mortality.

The objective of this study was to examine the association between CRF and the risk of (1) testing positive for COVID-19, and (2) dying from COVID-19, among participants of the UK Biobank study (UKB).

## Methods

This prospective cohort study has been conducted using the UK Biobank (UKB) Resource [19]. Research Ethics approval was received from the University of Toronto Research Ethics Board (REB) (protocol #39368). Individual participants provided written consent for their data to be shared with external researchers who have REB approval to access the UKB. Therefore, the specific research team for this project did not collect any consents. The UKB is a large population-based cohort that recruited approximately 500,000 adults aged 40 to 69 years from 2006 to 2010. In 2009, the UKB added a baseline CRF assessment, which 95,152 participants (18.9%) completed.

At baseline, questionnaires were used to capture demographic information (e.g., education, ethnicity) and medical history, while trained technicians measured height, weight, and conducted physical health assessments (e.g., CRF). Details of the recruitment process and data collection can be found elsewhere [19, 20].

On March 16^th^, 2020, the UKB was linked to COVID-19 testing data and results [21]. As of July 26, 2020, a total of 20,554 tests had been performed on 13,502 UKB participants; our sample represents the number of individuals tested who also completed the CRF assessment (n = 2,722). Participants were excluded if they were tested for COVID-19 posthumously (n = 3), if they were missing data on body mass index (BMI) (n = 4), alcohol use frequency (n = 12), or smoking status (n = 13). This left a total of 2,690 participants for analysis. Analyses examining risk of COVID-19 mortality included a sub-set of participants who tested positive for COVID-19 before the end of mortality data follow-up (July 1, 2020) (n = 346).

### CRF assessment

CRF was assessed by trained technicians using a submaximal bicycle test [22]. Each CRF assessment followed a similar pattern: (1) a pretest phase of 15 seconds; (2) a constant phase of 2 minutes where women received a workload of 30 W and men of 40 W; (3) the incremental phase which was 4 minutes. The incremental phase varied slightly among participants based on their responses to an initial questionnaire, which assigned them individuals to different protocols based on perceived risk Heart rate was captured throughout the test using a 4-lead ECG device (CAM-USB 6.5 with Cardiosoft v6.51 software). We used linear regression to estimate workload at the predicted maximum heartrate. Maximum oxygen consumption (VO_2_ max) was estimated using the formula: 7ml/min/kg + ((10.8ml/min/watt x predicted maximum workload in watts)/weight in kilograms) [23]. Estimated CRF (eCRF) was operationalized in two ways: first, participants were classified as having low eCRF (yes/no) if their estimated VO_2_ max was in the lowest 20^th^ percentile within their sex and 10-year age bands [14, 24]. Second, eCRF was classified as a three-category variable with participants being classified as having low (<20^th^ percentile), moderate (20^th^ to <80^th^ percentile), and high eCRF (≥ 80^th^ percentile) by sex and within 10-year age bands [24]. We also conducted a sensitivity analysis for the three-category eCRF variable where we classified individuals as having low (<20^th^ percentile), moderate (20^th^ to <60^th^ percentile), and high CRF (≥60^th^ percentile) by sex and within 10-year age bands [25].

### Outcomes

Individuals were classified as having COVID-19 if they tested positive at an out-patient or in-hospital laboratory between March 16, 2020 and July 26, 2020 by PCR test. The majority of specimen were taken in the nose or upper respiratory tract (94% or 2524 of 2690). Primary and underlying cause of death was obtained from the National Health Service Information Centre for participants in England and Wales, and the National Health Service Central Register for participants in Scotland through June 30^th^, 2020. Individuals were classified as having died from COVID-19 if their primary or underlying cause of death were ICD-10 codes U071 [COVID-19, virus identified] or U072 [COVID-19, virus not identified].

### Covariates

Age at testing was treated as a continuous variable, and calculated using age at UKB assessment, month of COVID-19 testing, and month of birth. Other demographic and lifestyle variables were self-reported at baseline and assumed to remain constant over the follow-up period. The following variables were considered as covariates: sex (male/female); race (white/Asian/Black/Other); education (secondary/post-secondary/missing); smoking (never/current/previous); alcohol use frequency (never/special occasions only/one to three times a month/once to twice a week/three or four times a week)/body mass index (underweight/normal weight[<25 kg/m^2^]/overweight [≥ 25 kg/m^2^ and <30 kg/m^2^]/obesity[≥30 kg/m^2^]).

Chronic conditions were assessed at baseline and updated over the follow-up period using in-patient hospital administrative databases and cancer registries. Evaluated chronic conditions included: immune disorders (e.g., whole organ transplant), cardiovascular disorders (e.g., high blood pressure), respiratory disease (e.g., chronic obstructive pulmonary disorders), liver disease, kidney failure, cancer, and diabetes (**Table 1**).

**Table 1:** Characteristics of the whole study population and those who tested positive for COVID-19.

Variable	Tested for COVID-19	Tested Positive for COVID-19
Sample size (n)	2,690	346
**DEMOGRAPHICS**		
Age (median (IQR))	70 (61, 75)	67 (57, 74)
Male (n (%))	1,314 (49.0)	180 (52)
**Race, n (%)**		
White	2,416 (90)	289 (84)
Asian	100 (4)	17 (5)
Black	104 (4)	25 (7)
Mixed/Other/Missing	70 (3)	15 (4)
**Education, n (%)**		
Secondary	535 (20)	65 (19)
Post-secondary	1,657 (62)	227 (66)
Missing	498 (19)	54 (16)
**ANTHROPOMETRIC MEASURES**		
Body Mass Index in kg/m^2^ (mean ± SD)	28.2 ± 5.2	28.9 ± 5.5
**Body Mass Index Category, n (%)**		
Underweight/Normal weight	758 (28)	83 (24)
Overweight	1,099 (41)	148 (43)
Obesity	833 (31)	115 (33)
**LIFESTYLE RISK FACTORS**		
**Smoke Status, n (%)**		
Never	1,048 (39)	151 (44)
Current	308 (11)	35 (10)
Previous	1,334 (50)	160 (46)
**Alcohol Use Frequency, n (%)**		
Daily	547 (20)	60 (17)
Three or four times a week	551 (20)	56 (16)
Once or twice a week	672 (25)	86 (25)
One to three times a month	280 (10)	43 (12)
Special occasions only	365 (14)	48 (14)
Never (n (%))	275 (10)	53 (15)
**Estimated Cardiorespiratory Fitness**		
Estimated VO_2_ max, mean ± SD	27.3 ± 5.5	27.3 ± 5.4
Three category, n (%)		
Low Fitness	529 (20)	77 (22)
Moderate Fitness	1,618 (60)	214 (63)
High Fitness	543 (20)	55 (15)
**CHRONIC CONDITIONS**		
**Immune System Disorders, n %**		
Whole Organ Transplant	170 (6)	22 (6)
HIV/AIDs	195 (7)	30 (9)
Inflammatory Disease of the CNS	27 (1)	8 (2)
Other immune system disorders	694 (26)	92 (27)
**Cardiovascular Disease, n (%)**		
Hypertension	1,244 (46)	169 (49)
Cholesterol Disease	1,131 (42)	147 (42)
Ischaemic Heart Disease	1,061 (39)	140 (40)
Pulmonary Heart Disease	1,254 (47)	169 (49)
Other forms of Heart Disease	1,116 (41)	151 (44)
Cerebrovascular Disease	1,129 (42)	155 (45)
**Respiratory Disorders, n (%)**		
Bronchitis/Emphysema	709 (26)	85 (25)
COPD	681 (25)	85 (25)
Asthma	807 (30)	105 (30)
Other Respiratory Disorder	794 (30)	98 (28)
Liver Disease, n (%)	1,387 (52)	173 (50)
Kidney Failure, n (%)	560 (21)	72 (21)
Diabetes, n (%)	498 (19)	76 (22)
Cancer, n (%)	554 (21)	69 (20)
Chronic Condition, n (%)	2,363 (88)	304 (88)
Number of Chronic Conditions, median (IQR)	5 (1, 8)	5 (1, 8)
COVID-19 Specific Death, n (%)	--	59 (17)

Abbreviations: IQR = interquartile range, % = percent, SD = standard deviation, CNS = central nervous system, COPD = chronic obstructive pulmonary disorder, COVID-19 = coronavirus-disease 19.

### Statistical analysis

Modified Poisson regression with log link was used to estimate adjusted risk ratios (aRR) and 95% confidence intervals (CI) for the association of eCRF with testing positive for COVID-19 and COVID-19 specific mortality. A forward model building approach [26] was undertaken separately for each outcome. Variables included as potential covariates are those described above. Given the well documented association between CRF and BMI [27–30], BMI was forced into all models. The final models for both testing positive for COVID-19 and COVID-19-related mortality included age at testing and BMI category, regardless of the eCRF variable used (i.e., binary low eCRF vs. three-level categorical variable). The models for testing positive for COVID-19 also included race, and the mortality models included sex. All analyses were conducted using SAS version 9.4. All tests were two-sided, and findings were considered statistically significant at an alpha of 0.05.

## Results

Characteristics of all participants are presented in **Table 1**. Approximately 13% of the sample (n = 346) tested positive for COVID-19, and there was a high case fatality rate of 17% (n = 59). Individuals who tested positive for COVID-19 were slightly younger than all those tested (median (IQR) = 67 (57, 74) versus 70 (61, 75)). All participants who were tested for COVID-19 had a high prevalence of comorbidities (median of 5 conditions) and approximately 88% had at least one chronic condition other than obesity. Approximately 31% of participants had obesity. However, participants were in the overweight BMI category on average (tested for COVID-19: 28.2 ± 5.2 kg/m^2^; tested positive: 28.9 ± 5.5 kg/m^2^).

There was no difference in the mean estimated VO_2_ max for those who were tested for COVID-19 and those who tested positive for COVID-19 (27.3 ± 5.5 ml/kg/min and 27.3 ± 5.4 ml/kg/min respectively). Most of the participants who were tested for COVID-19 had moderate fitness (n = 1,618, 60%), while approximately 20% had low (n = 529, 20%) and high fitness (n = 543, 20.2), respectively.

Compared to individuals with low eCRF, those with moderate (aRR = 0.93, 95% CI: 0.72, 1.21) or high (aRR = 0.77, 95% CI: 0.52, 1.15) eCRF were not at an increased risk of testing positive for COVID-19 (**Table 2**). Conversely, individuals with low eCRF had more than 2 times the risk of dying from COVID-19 compared to those with moderate or high fitness (aRR = 2.34, 95% CI: 1.35, 4.05). Further, when eCRF was categorized as high, moderate, and low, compared to individuals with low fitness, those with moderate fitness had a 57% (aRR = 0.43, 95% CI: 0.25, 0.75) lower risk and those with high fitness had a 63% (aRR = 0.37, 95% CI: 0.16, 0.85) lower risk of dying from COVID-19 (**Table 2**).

**Table 2:** Association between estimated cardiorespiratory fitness (eCRF) and testing positive for COVID-19 and COVID-19 mortality.

	Risk of Testing Positive	Mortality
	aRR (95% CI)^a^	aRR (95% CI)^b^
**ESTIMATED CARDIORESPIRATORY FITNESS**		
Low Fitness	1.08 (0.83, 1.41)	2.34 (1.35, 4.05)
**Three Category Variable**		
Low Fitness	Ref	Ref
Moderate Fitness	0.93 (0.72, 1.21)	0.43 (0.25, 0.75)
High Fitness	0.77 (0.52, 1.15)	0.37 (0.16, 0.85)

Abbreviations: aRR = adjusted relative risk, CI = confidence interval.

^a^Estimates are adjusted for age at testing, race (white, Asian, Black, Other), and BMI category (underweight/normal weight, overweight, obesity).

^b^Estimates are adjusted for age at testing, sex (male, female), BMI category (underweight/normal weight, overweight, obesity), and alcohol use frequency.

As a sensitivity analysis, eCRF was reclassified as low (<20^th^ percentile), moderate (20^th^ to <60^th^ percentile) and high (≥ 60^th^ percentile). Using this alternative classification, eCRF was still not a predictor of testing positive for COVID-19 (p = 0.41). The findings were also similar with regards to dying from COVID-19 in that individuals with moderate (RR = 0.42, 95% CI: 0.24–0.75) and high (RR = 0.44, 95% CI: 0.23–0.85) were at a significantly lower risk of dying from COVID-19 than individuals with low fitness.

## Discussion

This is the first study to examine the relationship between eCRF and COVID-19 infection and mortality. While we found that eCRF was not associated with risk of testing positive for COVID-19, we found evidence of a dose-response relationship wherein people with higher eCRF have a lower risk of dying from COVID-19.

Few population-based assessments of CRF exist, likely due to the higher technical and financial requirements of these tests compared to measured and self-reported physical activity^27^, limiting research into the impact of CRF on developing or dying from a communicable disease. Though partly heritable [31], measured and eCRF is also predicted by physical activity. It has been hypothesized that high CRF resulting from regular physical activity, especially exercise training, confers innate immune protection, attenuating the risk of infectious diseases including COVID-19 [18, 32]. However, the benefits of CRF in preventing COVID-19 may be complicated by the fact that participating in activities that promote CRF (e.g. physical or exercise in groups) could increase exposure to and spread of the virus. This paradoxical relationship may explain, in part, why we did not observe a significant association between CRF and testing positive for COVID-19.

Patients with severe COVID-19 may experience significant decreases in lung function, potentially requiring mechanical ventilation [33], and respiratory and circulatory failure are common causes of death among COVID-19 patients [34]. Since CRF measures the ability of these systems to supply oxygen to skeletal muscles during sustained activity, CRF may help identify individuals at the greatest risk for severe COVID-19 outcomes [17]. Consistent with this hypothesis, we found that low CRF more than doubled individuals’ risk of dying from COVID-19. In addition, having moderate to high CRF significantly decreased the risk of COVID-19 mortality, with high CRF reducing the risk of mortality even more than moderate CRF, suggestive of dose-response.

UKB participants are known to differ from the broader UK population. Previous research has suggested that the UKB is impacted by healthy volunteer bias, wherein UKB participants have better health and are less likely to live in socially deprived areas than the general UK population [20]. In the current study, approximately 88% of participants tested for COVID-19 in the UKB had at least one chronic condition other than obesity. This is consistent with an American study where 89% of patients hospitalized for COVID-19 had at least one underlying condition [35].

In addition, participants appeared to have a lower CRF than similar populations tested for COVID-19. Women in the current study had a comparable CRF to women in some [36], but not all studies. For example, CRF values for women in the current study were much lower than three cohorts from the United States and Norway (range: 30.4 to 34.4 ml/kg/min) [36–38]. Men had a lower CRF on average than men in all four of the above-mentioned studies (current study: 30.5 ± 5.2 ml/kg/min versus range: 33.8 to 42.6 ml/kg/min [36–38]). One possible explanation for these differences could be that while the cohorts directly measured CRF, in the current study CRF was estimated from a submaximal fitness assessment. Another possible explanation could be that testing was initially restricted to in-hospital symptomatic patients, such that those tested for COVID-19 would tend to have poorer health than the general public. As the risk of dying from COVID-19 was inversely associated with CRF, this could mean that the protective effects of CRF against COVID-19 mortality may be more pronounced in the general population.

This study has numerous strengths. First, exercise-based CRF assessments are rare, and the UKB uses trained technicians and a validated protocol to conduct the assessment. The comprehensive data available through the UKB ensured we had access to several important confounders, either self-reported or via linkage to administrative databases over the follow-up period. While previous studies of COVID-19 mortality have examined all cause-mortality, we had a sufficiently large sample to examine COVID-19-specific mortality, which is another important strength. Limitations of the current study include the approximate 10-year lag between baseline measurements (demographics, lifestyle variables, CRF assessment) and COVID-19 testing. We assumed that these variables remained constant over the follow-up period. As BMI and CRF are known to increase and decrease respectively with age, our results may be biased to the null. We attempted to mitigate this potential bias by categorizing BMI and CRF, as while these factors may change with age, they would be less likely to change so severely as to change categorizes. We also likely underestimated the true burden of comorbid conditions in this population as we only had access to hospital admissions and cancer registry data to capture diseases over the follow-up period.

In summary, this is the first study to examine the association between CRF and testing positive for or dying from COVID-19. Importantly, CRF was not significantly associated with testing positive for COVID-19 but having moderate or high CRF was associated with a significant decrease in the risk of dying from COVID-19. Physical activity is a modifiable behaviour that positively influences CRF [39] and has also been identified as a way to mitigate some of the potential negative health impacts from COVID-19 lockdowns [40–42]. This study provides additional support for these recommendations and suggests that prior physical activity could be protective against dying from COVID-19 among those that test positive.
